# E2DR: A Deep Learning Ensemble-Based Driver Distraction Detection with Recommendations Model

**DOI:** 10.3390/s22051858

**Published:** 2022-02-26

**Authors:** Mustafa Aljasim, Rasha Kashef

**Affiliations:** Electrical, Computer, and Biomedical Engineering, Ryerson University, Toronto, ON M5B 2K3, Canada; mustafa.aljasim@ryerson.ca

**Keywords:** deep learning, stacking, ensemble learning, distracted driving

## Abstract

The increasing number of car accidents is a significant issue in current transportation systems. According to the World Health Organization (WHO), road accidents are the eighth highest top cause of death around the world. More than 80% of road accidents are caused by distracted driving, such as using a mobile phone, talking to passengers, and smoking. A lot of efforts have been made to tackle the problem of driver distraction; however, no optimal solution is provided. A practical approach to solving this problem is implementing quantitative measures for driver activities and designing a classification system that detects distracting actions. In this paper, we have implemented a portfolio of various ensemble deep learning models that have been proven to efficiently classify driver distracted actions and provide an in-car recommendation to minimize the level of distractions and increase in-car awareness for improved safety. This paper proposes E2DR, a new scalable model that uses stacking ensemble methods to combine two or more deep learning models to improve accuracy, enhance generalization, and reduce overfitting, with real-time recommendations. The highest performing E2DR variant, which included the ResNet50 and VGG16 models, achieved a test accuracy of 92% as applied to state-of-the-art datasets, including the State Farm Distracted Drivers dataset, using novel data splitting strategies.

## 1. Introduction

With the continuous growth of the population, new technologies and transportation methods need to emerge to serve people effectively and efficiently. Building an efficient and safe transportation system can positively affect economies, the environment, and human mental and physical health. The increasing number of accidents is a major issue in our current transportation system. According to the World Health Organization, road accidents are the eighth highest reason for death worldwide [1]. According to [2], 1.35 million people die every year in a car accident, and up to 50 million people are injured. This makes traffic safety a major concern worldwide. More than 80% of road accidents are caused by distracted driving, such as using a mobile phone, talking to passengers, and smoking [2]. Therefore, more attention has been directed to driver action analysis and monitoring. Many efforts have been made to tackle the problem of driver distraction with effective approaches using countermeasures for distracted driver actions [3]. The measures can be divided into three categories: (1) distraction prevention before distraction occurs, (2) distracting action detection (through alertness) after distraction occurs, and (3) collision avoidance when a potential collision is expected [3]. Imposing strict fines, government legislation, and raising public awareness are methods used to decrease the number of accidents caused by distracted driving through preventing distraction sources before it happens. When a potential collision is expected, collision avoidance systems are implemented in most newly manufactured cars through lane control, automatic emergency braking, and forward-collision warning. Distraction alertness is critical and can be more effective in preventing driver distraction; thus, accurate real-time driver action detection methods are essential for this driver distraction category. Distraction alertness can be approached with different modalities. A camera was used to detect distracted driving behavior while driving in many applications. A Controller Area Network Bus can assess the vehicle’s performance, such as wheel angle and brake level. Moreover, the system can detect if the driver is distracted or focused on driving based on the in-car collected information. Finally, sensors, such as the electrocardiograph and the electroencephalograph, can be used to estimate the emotional and physiological states of a driver, which can be associated with the level of distraction and fatigue in drivers [4]; however, there is a lack of reflection on the fact that they are invasive sensors for a driver. The driver’s action or behavior, such as gaze, head pose, and hand position, can be detected through deep learning models and the analysis of the car information [5]. Existing methods in detecting driver distraction fall short in providing accurate detection and recommendations in real-time. Ensemble learning has shown better classification performance compared to individual models, as it combines the benefits of multiple models while overcoming their drawbacks [6]. While authors in [6] used a fixed architecture of only three deep learning models including the residual network (ResNet), the hierarchical recurrent neural network (HRNN), and the Inception network, there is a research gap in the literature in using a scalable and incremental stacking-based ensemble learning with real-time recommendations to achieve high accuracy in detecting distracted driving activities with minimal computational overhead. Thus, in this paper, we have proposed a novel scalable model that uses ensemble learning, focusing on stacking that combines two or more baseline models and generates an ensemble with better performance than the adopted models. Our method aims to enhance generalization, reduce overfitting, increase performance, and provide real-time recommendations. This paper first examines the performance of several state-of-the-art image deep learning classification methods. An Ensemble-Based Distraction Detection with Recommendations model is designed, namely (E2DR), with the goal of improving the accuracy of distracted behavior detection. In the proposed E2DR model, two or more deep learning models are aggregated in a stacking ensemble. A recommendation layer is also provided for real-time recommendations to drivers in each case of the distracted behaviors to allow drivers or autonomous vehicles to take the best course of action when drivers are detected under distracted behaviors. Experimental results show that state-of-the-art image classification models achieve a test accuracy ranging between 82–88% in detecting driver distraction. Furthermore, results show an average improvement of 5–8% in detection accuracy when the proposed E2DR is used with a real-time data splitting based on the driver IDs. Similar results are obtained for other metrics such as Precision and F1 score. The rest of this paper is as follows: Section 2 discusses deep learning driver distraction detection systems and the related work. Section 3 and Section 4 introduce the adopted and proposed models, respectively. Section 5 presents the experimental results and analysis. We conclude the paper with future directions in Section 6.

## 2. Related Work on Deep Learning Driver Distraction Detection

There are three main types of distracted driving: cognitive, visual, and manual distraction. Cognitive distraction occurs when the driver’s mind is not entirely focused on driving. Driver gaze and talking to passengers are examples of cognitive distraction. Even drivers listening to music or the radio are at risk. The audio or music can shift the driver’s attention from driving and overall surroundings. Visual distraction is when the driver is not looking at the road ahead. Drivers who observe shop signs and billboards on the side of the road are considered visually distracted. Looking at electronic devices such as GPS devices, smartphones, and digital entertainment devices while driving is under the category of visual distraction. Finally, manual distraction occurs when the driver, for any reason, takes their hands off the steering wheel. Drivers who smoke while driving, eat and drink in the car, or try to get something from anywhere in the vehicle are under the risk of manual distraction. Texting while driving is the most dangerous driver distraction as it combines all three types of distractions. When drivers take their eye off the road to send a message or check a notification, it is long enough to cover the length of a football field at 80 km/h [5]. Various research studies have been proposed to address the drivers’ distraction detection problem. This section will survey the most recent state-of-the-art research work using Single-based vs. Hybrid-based deep learning to address this problem.

### 2.1. Single-Based Deep Learning Models

A gaze estimation model called X-Aware is introduced in [7] to analyze the driver’s face along with contextual information. The model visually improves the fusion of the captured environment of the driver’s face, where the contextual attention mechanism is directly attached to the output of convolutional layers of the InceptionResNetV2 networks. The accuracy of their best model outperformed the other baseline models in the literature. The dynamics of the driver’s gaze and their use to understand other attentional mechanisms are addressed in [8]. The model is built based on two questions, where and what is the driver looking at. The model is trained through coarse-to-fine convolutional networks on short sequences from the DR(eye)VE dataset [8]. Their experiments showed that the driver’s gaze could be learned to some extent, considering its highly subjective challenges and the scene’s irreproducibility showing the driver’s gaze for each sequence. The results showed that the model could achieve accurate results and could be integrated into practical applications. In [9], the authors proposed a deep learning model that detects drivers’ behavior and actions during travel. The deep learning model classifies the driver actions into ten classes. The first class represents safe driving, and the other nine classes represent unsafe drivers’ actions such as fixing makeup and texting. The driver receives an alert if an unsafe action is detected. They used Convolutional Neural Networks (CNNs) to perform training and detection. The core of the deep learning system is ResNet50. A dense net architecture followed the ResNet50 architecture to make classifications. The dataset used is the State Farm dataset and included images of different drivers’ actions that cause distracted driving. The model achieved high accuracy in detecting the driver’s actions. A facial expression recognition model in [10] monitors drivers’ emotions and operates in low specification devices installed in cars. A Hierarchal Weighted Random Forest Classifier (HRFC) is used and trained on the similarity of sample data. Geometric features and facial landmarks are detected and extracted from input images. The features are vectorized and implemented in the Hierarchal Random-Forest Classifier to detect facial expressions. The method was evaluated on the MMI dataset, the Keimyung University Facial Expression of Drivers (KMU-FED) dataset and the Cohn-Kahnde dataset. The results showed that the proposed model had similar performance to other state-of-the-art methods. The study in [11] introduced a computationally efficient distracted driver detection system based on convolutional neural networks. The authors proposed a new architecture called mobileVGG. The architecture is based on depth-wise separable convolutions. The authors used a simplified version of the VGG16 model, allowing the proposed architecture to be suitable for real-time applications with decent classification accuracy. The datasets used are the State Farm dataset and the American University in Cairo Distracted Driver (AUCDD) detection dataset. The results showed that the proposed architecture outperformed other approaches while being computationally simple. The driver’s face pose is detected by training CNNs [12]; the CNNs then identify if the driver’s head position is considered under the category of distracted driving. The model consists of five CNNs followed by three fully connected layers. The results showed that the proposed model has better accuracy when compared to non-linear and linear embedding algorithms. In [13], the authors proposed a driver action recognition system called dilated and deformable Faster Region-Based Convolutional Neural Networks (R-CNN). It detects driver actions by detecting motion-specific objects exhibiting inter-class similarity and intra-class differences. The irregular and small features, such as cell phones and cigarettes, are extracted through the dilated and deformable residual block. Then, the region proposal optimization network algorithm decreases the number of features and improves the model’s efficiency. Finally, the feature pooling module is replaced with a deformable one, and the R-CNN network is trained as the classifier of the network. The authors established the dataset and contained images of different driver actions. Results showed that the model demonstrates acceptable results. Authors in [14] implemented a driver distraction detection model that uses a light-weight octave-like convolutional neural network. The network consists of octave-like convolutional blocks called OLCMNet. The OCLM block splits the feature map into two branches through point-wise convolution. Average pooling and depth-wise convolution are performed on the feature map. A DC operator captures the fine details in the high-frequency branches. Lastly, the OCLMNet exchanges further information between layers. The model performed well on the Lilong Distracted Driving Behavior dataset while being implemented on a limited computation budget. A unique approach is proposed in [15] that uses both spatial and temporal information of electroencephalography (EEG) signals as an input to a deep learning model. The relationship between the driver distraction and the EEG signal in the time domain is mapped through gated recurrent units (GRUs) and CNNs. Twenty-four volunteers were tested while doing activities that cause a distraction while driving, and their EEG response was recorded. Then, the proposed deep learning network was trained based on the EEG information. The deep learning approach consisted of a temporal–spatial information network (TSIN), combining CNNs and GRUs to better detect spatial and temporal features from EEG signals. The authors of [16] proposed a modifier deep learning approach for distraction detection. They used the OpenPose library for a two-category problem of distraction detection. The library draws 43 points on the facial skeleton to detect the human face. The detection is sent to a deep neural network that uses the ResNet50 model. The results demonstrated good accuracy and outperformed other residual network architectures. The work in [17] introduced a new approach to distracted driver detection using wearable sensing and deep neural networks. The study included information from twenty participants through wearable motion sensors attached to their wrists. The participants performed five distraction activities under instructions in a driving simulator. The captured data were sent to a deep learning model that consisted of recurrent neural networks and long short-term memory (RNN-LSTM) which classified distraction tasks. The results showed a good potential for the wearable proposed sensing approach.

### 2.2. Hybrid-Based Deep Learning Models

A hybrid model is designed in [18] with an ensemble of weighted CNNs for driver posture classification. The authors proved that using a weighted ensemble classifier using the genetic algorithm resulted in a better confidence score for classification. Additionally, the effect of variable visual elements is analyzed, such as face and hands, in detecting distracted driver action through localization of face and hands. The dataset used is the Distracted Driver dataset, which contains ten classes of driver actions. The best model has an accuracy of 96%, and a smaller version of the ensemble model achieved an accuracy of 94.29%. In [19], a distracted driver detection technique using pose estimation is introduced. The model is an ensemble of ResNets and classifies drivers through pose estimation images. ResNet and HRnet are used to generate pose images. Then, ResNet50 and ResNet101 classify the original and pose estimation images. The grid search method identifies the optimum weight for predictions from both models. Classifying pose estimation images is useful when used with the original image classification model as it increases classification accuracy. The dataset used is the AUCDD dataset. The results showed that the introduced model achieved an accuracy of 94.28%. The study in [20] detected driver–vehicle volatilities using driving data to detect the occurrence of critical events and give appropriate feedback to drivers and surrounding vehicles through analyzing multiple real-time data streams such as vehicle movements, instability of driving, and driver distraction. The deep learning model consisted of a Convolutional Neural Network (CNN) model and a long short-term memory (LSTM) model. The data were collected from more than 3500 drivers and included 1315 severe and 7566 normal events. The model achieved high accuracy and was effective in detecting accidents. A CNNs-based model to identify driver’s activities is introduced in [21]. The driving activities are divided into several classes, in which 4 are considered normal driving activities, and the other three are classified as distracted driving. The Gaussian mixture model detects the driver’s body from the background before sending the image to the CNN model. The authors used transfer learning to fine-tune three pre-trained state-of-the-art CNN models: Restnet50, AlexNet, and GoogLeNet. The model was trained as a binary classifier to detect whether the driver was distracted or not. The authors collected the data from 10 drivers involved in the most common driving activities. The results showed that the model was effective as a binary classifier. In [22], a model for distracted driving action recognition is proposed using a hybrid of two convolutional neural network architectures, Xception and Inception V3, to detect 10 classes of driver actions. The authors used ImageNet weights for transfer learning. The performance of both models was analyzed under different weighting schemes. Using pre-trained weights helped the network learn basic shapes and edges without starting training from scratch, which allowed the model to achieve good results in under 10 epochs of training, applied to the State Farm Distracted Driver dataset. The results showed that the Inception model had a better performance compared to the Xception model. A distracted driver action recognition system is introduced in [23] based on the Discriminative Scale Space Tracking (DSST) and Deep Predictive Coding Network (PCN) algorithms, dynamic face tracking, location, and face detection. Then, the YOLOv3 object detection model detects distracting behavior around the driver’s face, such as phone calls and smoking. The dataset used is a self-built dataset of people making phone calls and smoking. The results demonstrated that the model could detect a driver’s behavior with high accuracy. A hybrid driver distraction detection model is presented in [24]. The model uses CNNs and Bidirectional Long Short-Term Memory (BiLSTM). The proposed model captures the spatio-spectral features of the images and consists of two steps: (1) detect the spatial features of different postures using CNNs automatically, and (2) the spectral components from the stacked feature map are extracted through the BiLSTMs. They used the AUCDD dataset. Results showed that their model performed better than most state-of-the-art models. The work in [25] used deep learning to detect driver inattentive and aggressive behavior. They classified inattentive driver behavior into driver fatigue, downiness, driver distraction, and other risky driver behavior such as driving aggressiveness. All these risky driving behaviors are associated with various factors that include driving age, experience, illness, and gender. The authors used CNNs, RNNs and LSTMs. They showed that the CNNs achieved the best performance. The algorithm in [26] detects driver manual distraction using two modules; in the first module, the bounding boxes of the driver’s right ear and right hand are detected from RGB images through YOLO, a deep learning object detection model. Then, the bounding boxes are taken as an input by the second module, a multi-layer perceptron, to predict the distraction type. The dataset consisted of 106,677 frames extracted from a video obtained from 20 participants in a driving simulator. The proposed algorithm achieved comparable results with other models in the same field. Table 1 provides a comparative study of the recent work in driver distraction detection systems. There is a research gap in the literature in using stacking-based ensemble learning to achieve high accuracy in detecting distracted driving activities with minimal computational overhead. Thus, in this paper, we have proposed a framework that uses ensemble learning, focusing on stacking that combines two baseline models and generates an ensemble with better performance than the adopted models.

## 3. Adopted Deep Learning Models

### 3.1. ResNet50 Model

**Figure 1:**
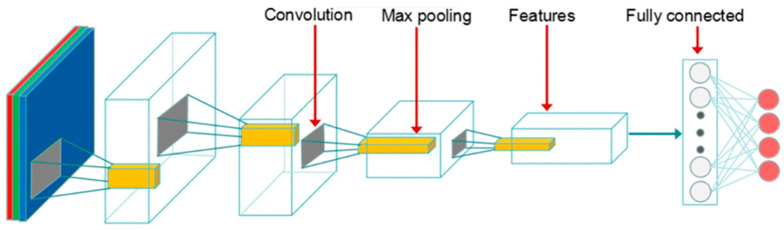
The ResNet50 blocks [27].

ResNet50 is a 50-layer deep convolutional neural network (Figure 1) [27]. The pre-trained version of the network can be imported from the ImageNet database [28], trained on over a million photos. The network is trained to classify images into 1,000 different object categories, including pencils, tables, mice, and various animals and objects. As a result, the network has learned a variety of rich feature representations for a large variety of images [29]. In the final classification model, ResNet50 is used as the convolutional base, and the pre-trained model is used to learn the patterns in the data. ResNet50 requires the size of the input images to be 224 × 224 (width, height) [29]. All the experiments were conducted with color images. The dimensions of each image sent to the classification model were (224, 224, 3), where 3 indicates the number of channels in the images. The three channels indicated are color channels composed of Green, blue, and red.

### 3.2. VGG16 Model

**Figure 2:**
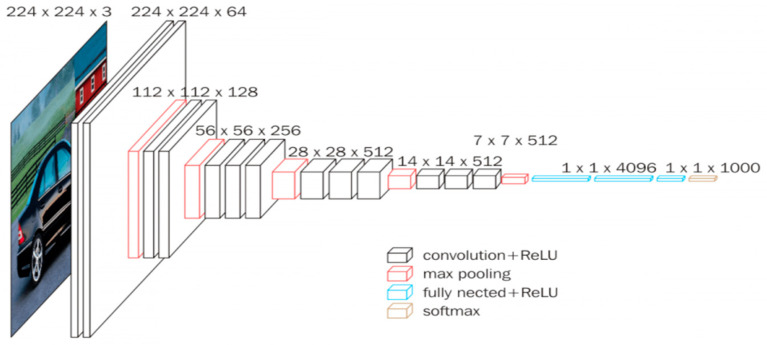
The VGG16 blocks [33].

Oxford’s VGG16 architecture from the Visual Geometry Group (VGG) (Figure 2) has an advantage over AlexNet by replacing large kernel-sized filter 5 and 100 in the second and first convolutional layers, respectively, with several kernel-sized filters of size 3 × 3 one after another [30]. Similar to the ResNet50 model, the input to the network is a 224 × 224 × 3 image, where 3 indicates the RGB channels. The image is passed through a series of convolutional layers with small receptive field filters with a size of 3 × 3 [31]. This filter size is the smallest to capture center, up/down and left/right notions. Additionally, the configuration utilizes 1 × 1 convolutional filters that can be considered a linear transformation of the input channel [32].

### 3.3. Inception Model

**Figure 3:**
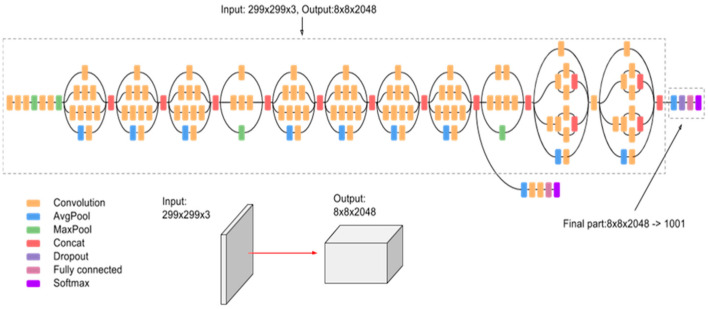
The Inception v3 architecture [39].

The Inception model (Figure 3) is a significant milestone in the evolution of CNN classifiers. Inception changed the traditional approach of adding more and deeper convolutional layers to improve performance [34]. Inception went through several versions and developed with time. Inception V1 [35] uses multiple filters that operate simultaneously, making the network wider rather than deeper. Then, the authors introduced Inception V2 and V3 [36]. Inception V2 significantly improved performance and computational speed by using two 3 × 3 convolutional operations instead of a single 5 × 5 convolution which is 2.78 times more computationally expensive [37]. Inception V3, the model used in the experiment, includes all upgrades in Inception V2 and factorized 7 × 7 convolutions, which improved performance even more, and added Label Smoothing to decrease the chances of overfitting [38]. The main contribution of Inception V4 is adding reduction blocks to change the width and height of the grid [39].

### 3.4. MobileNet Model

**Figure 4:**
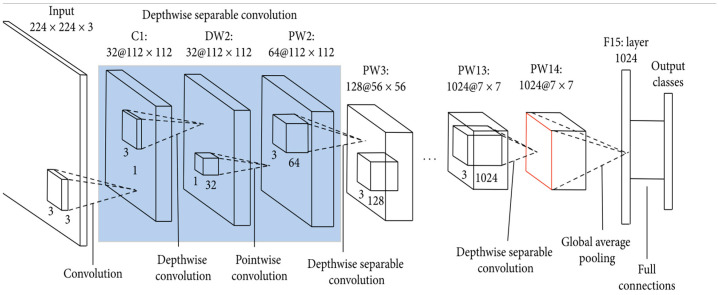
The MobileNet Architecture [43].

MobileNet (Figure 4) is the first mobile computer vision model based on TensorFlow [40]. The name Mobile implies the ability of the model to function in mobile applications [41]. MobileNet is based on separable depth-wise convolutions, which significantly reduces the number of parameters, especially when compared to networks with regular convolutions that have the same depth of the nets. This makes MobileNet a lightweight deep neural network suitable for mobile applications [42]. The depth-wise separable convolution is made from two primary operations: depth-wise convolution and point-wise convolution. The depth-wise convolution came from the idea that the filter’s spatial and depth dimensions can be separated. The filter is separated by its height and width dimensions, and then the depth dimension is separated from the horizontal (width×height) dimension. The point-wise convolution is a 1 × 1 convolution that changes the dimension of the previous layer.

## 4. The Proposed E2DR Model

Existing driver distraction detection systems in the literature only use a single model trained for classification. Moreover, most recent work uses a single state-of-the-art classifier or a network of convolutional neural layers to get the best performance. In this paper, an Ensemble-Based Distraction Detection with Recommendations system is designed, namely E2DR, to improve the accuracy of driver distraction detection and provide recommendations. In the proposed E2DR model, two deep learning models are aggregated in a stacking ensemble. A recommendation layer is also provided for real-time recommendations in each case of distracted behaviors. The E2DR model enhances the generalization of the detection process and reduces overfitting. The E2DR model allows drivers or autonomous vehicles, depending on the technology in the vehicle, to take the best action when drivers are detected under distracted behaviors.

### 4.1. The Ensemble-Based Distraction Detection with Recommendations Model (E2DR)

**Figure 5:**
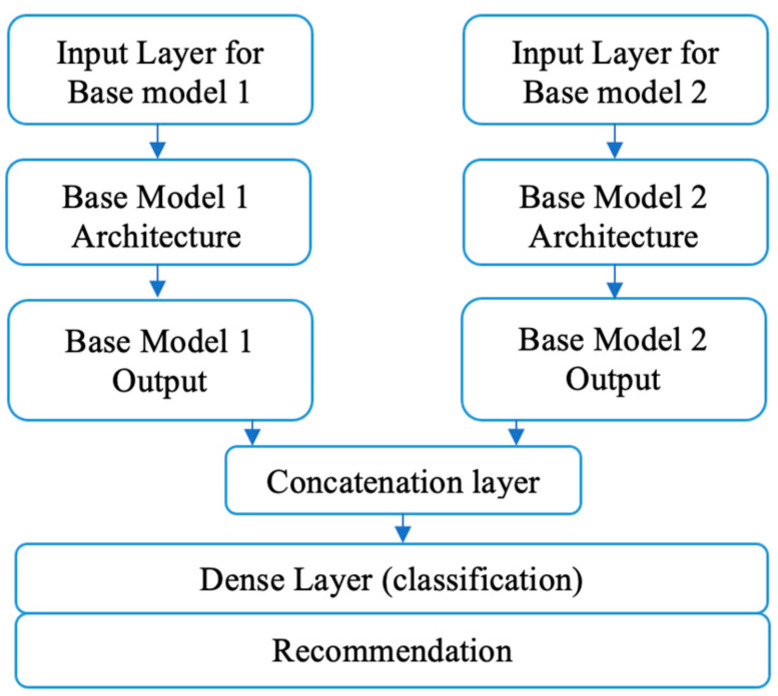
The E2DR (Pair-wise stacking) Architecture.

Stacked generalization (SG) was first introduced in [44]; it was shown that stacking reduces the bias of the single model concerning the training set, where bias is the average difference between actual and predicted results [44]. The deduction results from the stacking model’s ability to harness the capabilities of more than one well-performing model on a regression or classification task to generate better performance and predictions than base classifiers in the ensemble, which reduces the error and bias. Inspired by the theory of stacking generalization, the E2DR model combines two or more detection models to provide better and more accurate predictions than individual models. The stacking algorithm takes the output of the base model as an input to another model, sometimes called a meta-learner, which learns how to combine the predictions of base models to generate better predictions. In more detail, the stacking architecture has two levels. The first level contains the base models, and the second level includes a meta-learner that concatenates the outcomes of base models to provide final predictions. Figure 5 shows the pair-wise stacking in the E2DR architecture (i.e., only two base models are combined).

### 4.2. E2DR Variants

In this paper, six models are developed using variants of base model 1 and base model 2, such as E2DR (A1, A2) where A1 and A2 ∈ {ResNet, VGG16, MobileNet, Inception}. The E2DR model combines 2 of the mentioned models using the Stacked Generalization (SG) ensemble method. The SG ensemble method uses the outputs from the pre-trained base models, concatenates them, and sends them to a meta-learner model at level 2 consisting of a dense layer for classification. Once the distracted behavior is classified, a set of recommended actions are provided to ensure safety. We calculate an assessment measure for each E2DR (A1, A2) using Accuracy, Loss, F-measure, Precision, and Recall.

### 4.3. Computational Complexity

Assume T_A1_ and T_A2_ are the computational time taken by both base models A1 and A2, respectively. Assume the meta learner needs overhead of T_M_ and the recommendation layer needs O(k) to retrieve recommendations based on the output classification, where k is the number of recommendations. We assume a linear search algorithm for recommendations extraction. The overall computational complexity of the E2DR model is computed as the maximum of T_A1_ and T_A2_ in addition to the overhead of concatenation and recommendation retrieval, as shown in Equation (1).

T_E2DR_ = O(*Max*(T_A1_, and T_A2_)+ T_M_ +O(k) (1)

### 4.4. Adopted Base Model Architectures

All adopted architectures use CNNs, a deep learning model that learns from spatial features of images by creating feature maps using filters and kernels (sliding windows). Many variations of CNNs have been studied recently to detect driving postures and actions. The models adopted in this paper are among the highest-performing CNN models. For all models, we used a learning rate of 0.001 and Categorical Cross Entropy as the loss function. The models are explained in detail in Section 3.

**Figure 6:**
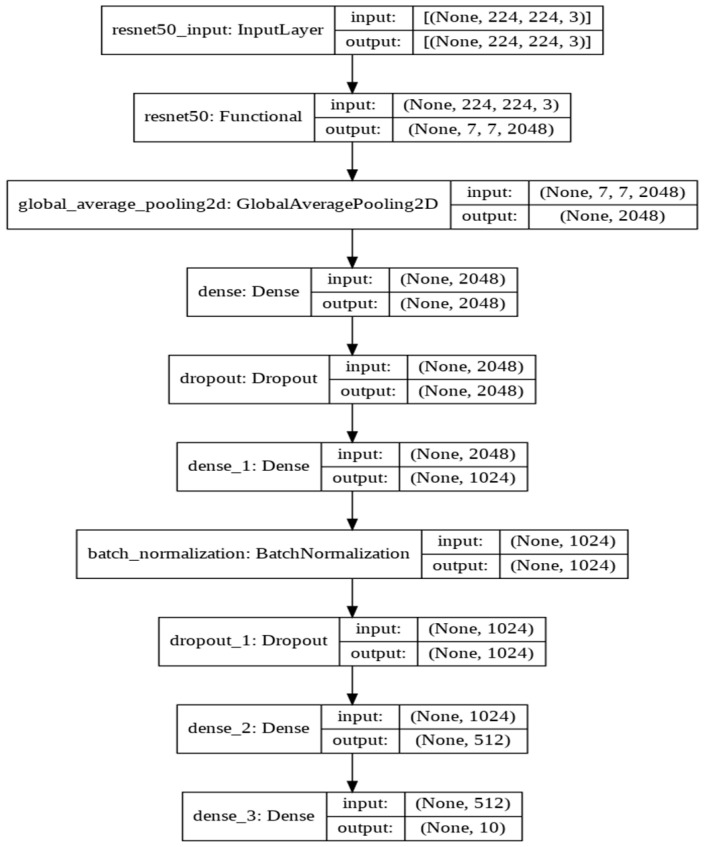
ResNet50 Architecture.

*ResNet50 Model:* When building the CNN model, the classification top of the ResNet50 Model (Figure 6), which was originally designed to classify 1000 classes [45], was dropped from the network to adapt the new CNN architecture to the dataset used that included only 10 classes. To avoid the problem of overfitting, a drop-out layer was added, and performance on the validation set was observed after every epoch. The hyperparameters, such as batch size, learning rate, and the number of neurons, were adjusted to optimize the model’s performance and enhance the model’s generalizability. We used different batch sizes: 128, 64, 32, and 16 to understand the significance of selecting the batch size as well as its effect on the network, with 32 as the best performing batch size.

**Figure 7:**
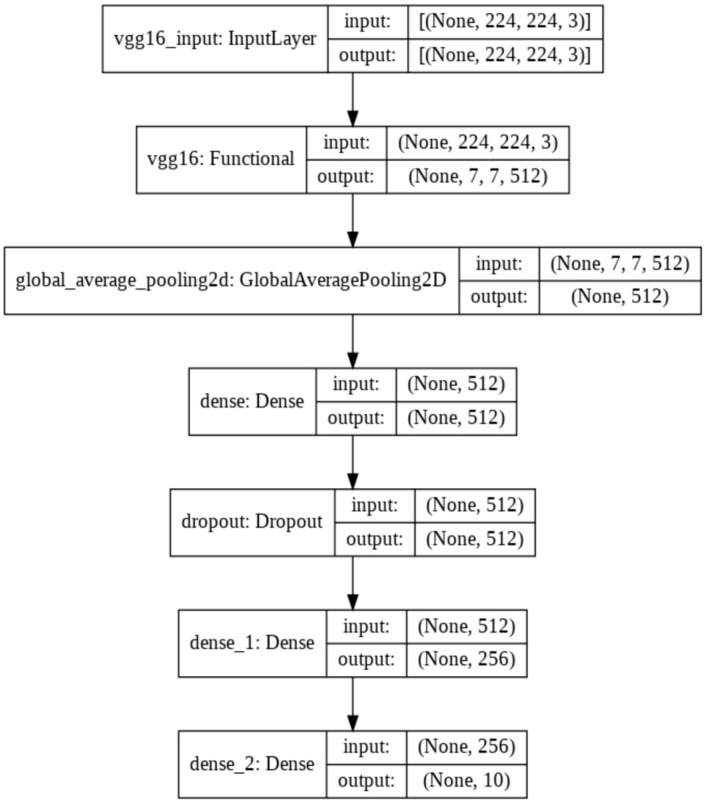
Dense net architecture (VGG16).

*VGG16 Model*: The VGG16 model (Figure 7) is among the largest models with many parameters. The padding is 1 pixel for 3 × 3 convolutional layers to preserve the spatial padding after undergoing convolution. The five-max pooling layers carry out the spatial pooling that follows some convolutional layers [46]. The max pooling is applied through a 2 × 2 pixel widow that has a stride value of 2. The same filter size is applied several times, allowing the network to represent complex features [47]. This “blocks” concept became more common after VGG was introduced [46]. As with the ResNet50, the classification layer (top layer) is adapted to the used dataset with 10 classes. The hyperparameter was chosen to optimize performance and training time with a batch size equals to 32.

**Figure 8:**
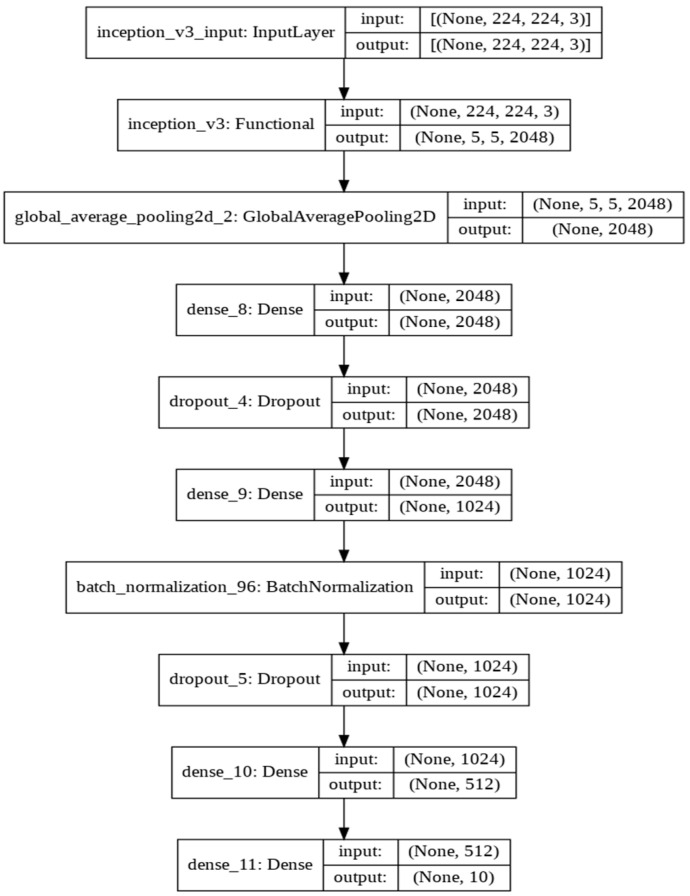
The Inception Model Architecture.

*Inception model:* The Inception model (Figure 8) is a widely-used image classification model with medium complexity. Its continual evolution resulted in the creation of multiple versions of the model. The one used in the experiments is the third version of the network, which has similar parameters to the ResNet50 model discussed earlier. The batch size equals 32, and the model is trained for 5 epochs. The model uses smaller convolutions which can be significant in decreasing computational time. In addition, factorizing convolutions reduces the number of parameters. Therefore, the Inception model has a lower training time compared to the ResNet50 and VGG16 models.

**Figure 9:**
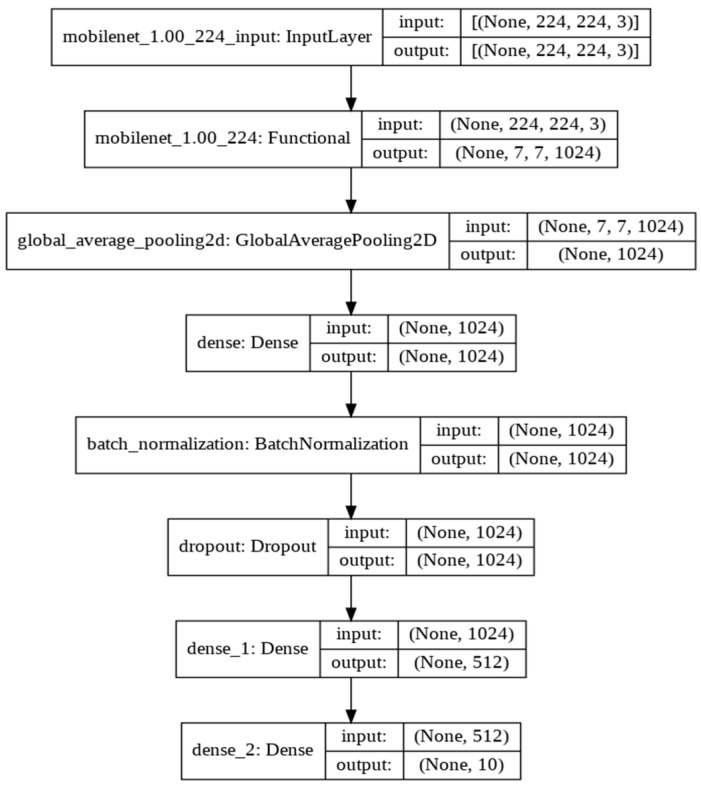
Model Architecture (MobileNet).

*MobileNet model:* we used the MobileNet architecture (Figure 9) to classify drivers’ actions to examine its performance, considering it is the smallest state-of-the-art image classification network in terms of size and number of parameters. The main difference compared to other models is that MobileNet uses a 3 × 3 depth-wise convolution and 1 × 1 point-wise convolution instead of the traditional 3 × 3 convolution layer in most CNN models. The dense network is similar to the ResNet and Inception model networks discussed earlier. The batch size is 32, and the model was trained for 5 epochs. The top layer was removed from the network as with other models to modify the classifications according to the dataset used.

### 4.5. Recommendations

Adding a recommendation after the classification of each class can be a great improvement to the distracted driver detection implementations. In most cases, the best action to consider, especially in driving, is as easy as keeping the driver’s focus on the road with no distraction. Reminding drivers is the best action to consider avoiding losing their attention during driving. An alert system can be effective with alternatives and actions to remind drivers of options to ensure their safety, especially drivers that need to take an important phone call or send an urgent text. In the case of autonomous vehicles, the recommendations can be sent to the vehicle to perform the best course of action. However, this is limited by the technology available in the vehicle. Table 2 provides the list of recommended actions as alertness signals to the driver based on the class of distracting activities while driving. The category of the distracting activities is retrieved from the ensemble classification layer in the E2DR model.

## 5. Experimental Analysis and Results

### 5.1. Dataset

**Figure 10:**
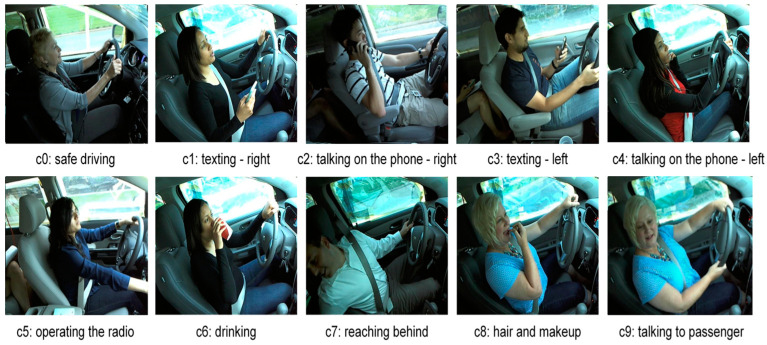
State Farm Distracted Driver dataset class representation [47].

The dataset used in the experiment is the State Farm Distracted Drivers dataset [48]. There are 22,424 images of drivers in distracted positions that can be used for training and testing. The images in the dataset were taken with the contribution of 26 unique subjects in different cars (random numbering (i.e., not in consecutive order)). The dataset has 10 classes: safe driving, texting—right, talking on the phone—right, texting—left, talking on the phone—left, operating the radio, drinking, reaching behind, hair and makeup, and talking to a passenger. A representation of each class in the dataset is shown in Figure 10.

### 5.2. Experimental Setup

A MacBook Pro and Google collab (Pro) are used to train and test the models.

### 5.3. Preprocessing and Splitting Strategy

The Distracted Driver dataset is ingested, then preprocessed as follows: (1) the images and the driver_imgs_list.csv file are stored, (2) the images are loaded, converted from BGR to RGB, and resized to 244 × 244 × 3 as this is the size used by most models and architectures for transfer learning, (3) the data are split into training, validation, and test sets; the validation and test set was created based on the subject (Driver ID); the subjects chosen for validation were: p18, p27, and p39, and the subjects chosen for testing were: p015, p022, p050, and p056. Finally, the labels were converted into categorical values. As a result, the training set had 15,963 images, the validation set had 2769 images and the test set had 3692 images.

### 5.4. Evaluation Metrics

Precision, Recall, F1 score, and Accuracy [49,50,51,52,53,54] are the most well-known evaluation metrics to assess the performance of a classifier. Precision finds pertinent instances among the gathered instances. It can be defined as the ratio between the True Positives (TP) and the sum of True Positives and False Positives (FP) as shown in Equation (2).

(2) Precision=(TP)/(TP+FP)

Recall, also known as Sensitivity, is defined as the ratio of True Positives and the sum of True Positives and False Negatives (FN).

(3) Recall =(TP)/(TP+FN)

F1 Score: It is defined as the harmonic mean of Precision and Recall as shown in Equation (4).

(4) F1−Score =(2 ∗ Precision × Recall)/(Precision+Recall)

Accuracy finds the correct predictions among the total predictions. It is defined as the ratio between the sum of True Positives and True Negatives and the sum of True Positives, False Negatives, True Negatives, and False Negatives.

(5) Accuracy =(TP+TN)/(TP+FP+TN+FN)

### 5.5. Performance Evaluation: Base Models

**Figure 11:**
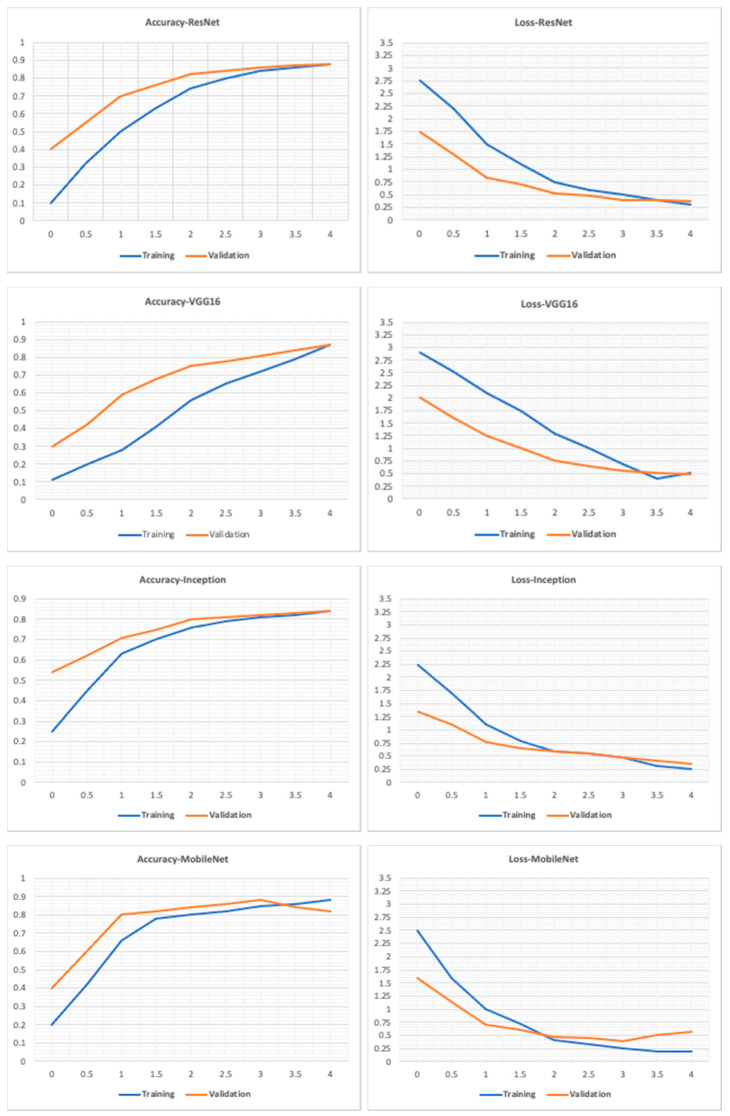
Accuracy and loss graphs for the models on the training and validation sets.

**Figure 12:**
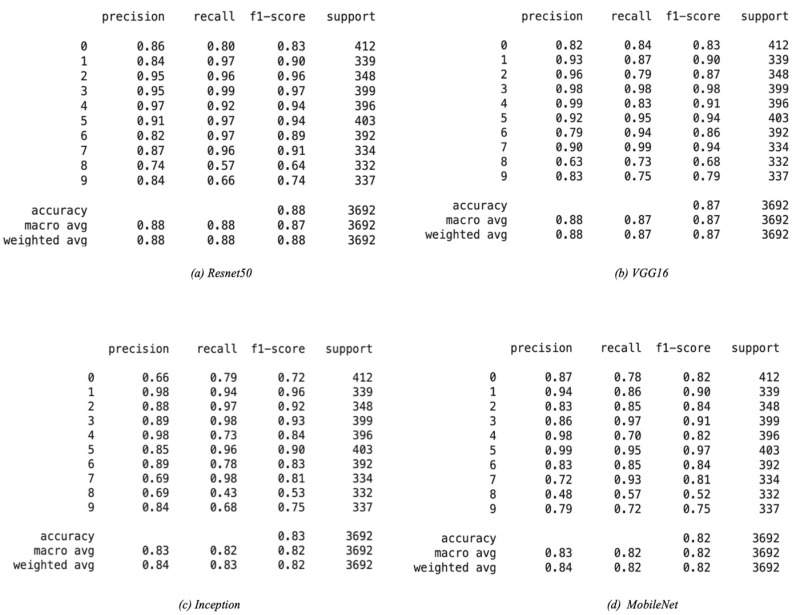
Performance reports for deep learning models on the test set.

**Figure 13:**
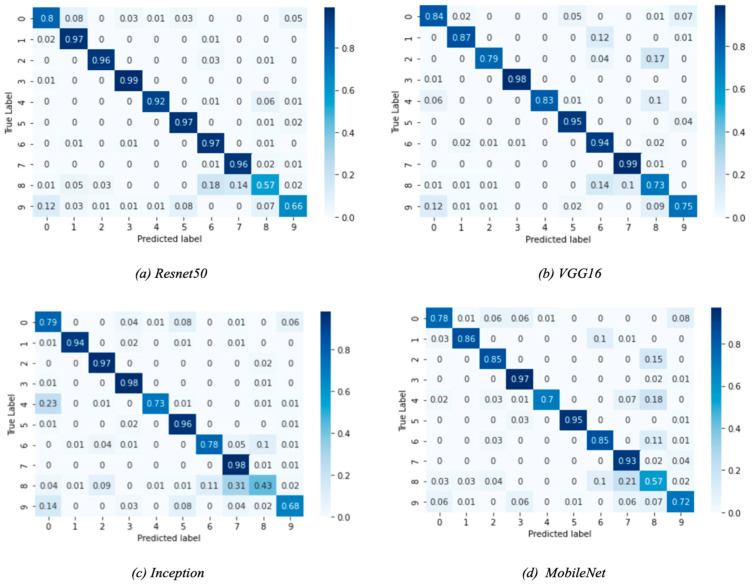
Confusion matrices for the deep learning models.

The performance of all base models is shown in Table 3. ResNet50 performs best with the highest accuracy and recall on the test set. The VGG16 performs just as well as the ResNet50 model, but the training time was significantly longer than ResNet50 and all other tested models since it has many variables. The Inception model had a test accuracy of 0.83, lower than ResNet50 and VGG16. The Inception model had the highest loss, and the training time was close to the ResNet50 model. Finally, the MobileNet had a similar performance to the Inception model with a lower loss and significantly faster training time. This is because MobileNet is a low-power, low-latency, light model parameterized to meet the constraints of computational and time resources of various applications. Choosing which optimum model to use depends on the trade-off between performance and computational complexity. ResNet50 and VGG16 can be used when powerful and robust computational resources and flexible time constraints are available. The MobileNet can be used for faster training and decent accuracy, which is not as good as VGG16 and ResNet50 but is still a good choice for limited computational resources. The Inception model did not perform well and lagged other models in most aspects, making it not favorable compared to the other tested models. Figure 11 shows how each model performed on the training, validation, and test sets. As shown in Figure 11, all models have a gradual increase in validation accuracy with the increasing number of epochs except for the MobileNet model, which has the highest validation accuracy at the third epoch and decreases afterwards. Similarly, the loss of all models decreases as epochs increase except for the MobileNet, which had the lowest loss value at epoch number three, which shows that the model was overfitting after epoch three. We even increased the number of epochs up to one, and we observed that the individual baseline models suffered from overfitting and could not show an increase in performance even when training was continued for a larger number of epochs. The performance report (Figure 12) shows how each model performs for each class. All models perform well for classes 1–5 and perform poorly for class 8, which is “hair and makeup”, because it is a challenging class to be detected and usually confused with class 7, as shown in the confusion matrices in Figure 13. Moreover, the data quality for this class might not be on the same level as other classes.

### 5.6. The E2DR Performance Evaluation

#### 5.6.1. Settings

In the first layer of the E2DR model, the individual models were trained. Their layers are executed in parallel (after optimizations) when loaded in the ensemble, so their weights are not altered during training. Each of the two base models has 10 outputs representing each class in the dataset. The output of the base models is sent to a concatenation layer, which is then sent to a dense layer with 10 neurons (equal to the number of classes). A SoftMax activation function is used to perform classification. A learning rate of 0.001 and Adam optimizer are used to compile the model with a batch size equal to 32. The loss function used is the Categorical Cross Entropy loss function. The E2DR was trained for five epochs similar to base models.

#### 5.6.2. Results and Discussion

**Figure 14:**
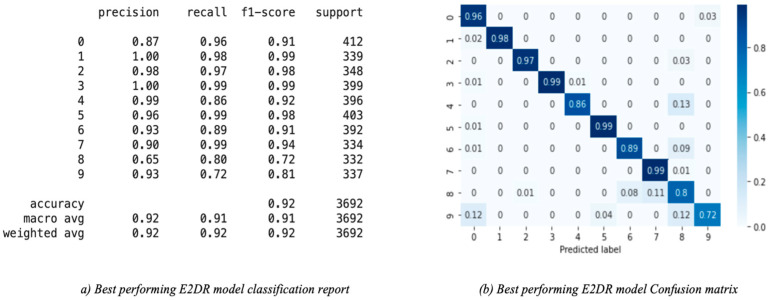
Best performing E2DR model classification report and confusion matrix.

**Figure 15:**
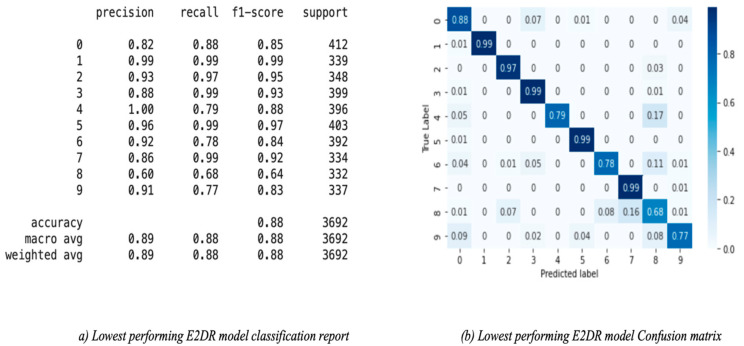
Lowest performing E2DR model classification report and confusion matrix.

**Figure 16:**
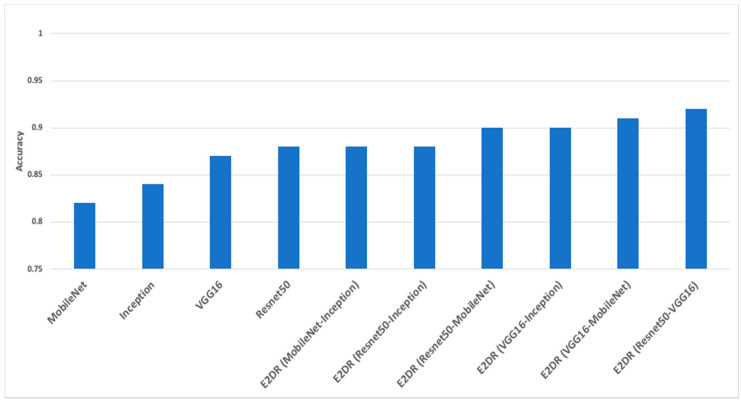
Accuracy of base models and E2DR models.

**Figure 17:**
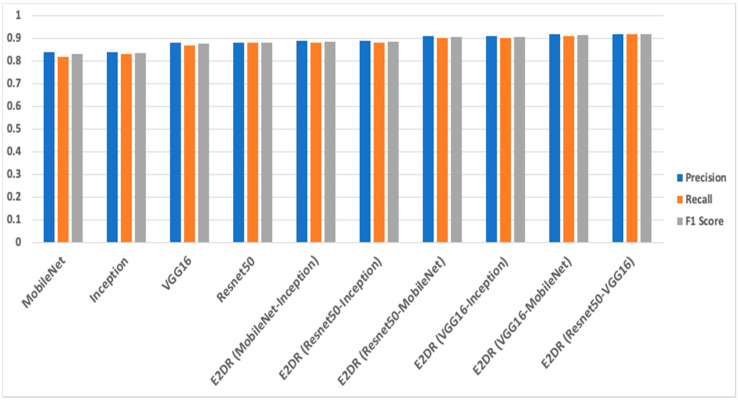
The Precision, Recall, and F1 Score of base models and E2DR models.

**Figure 18:**
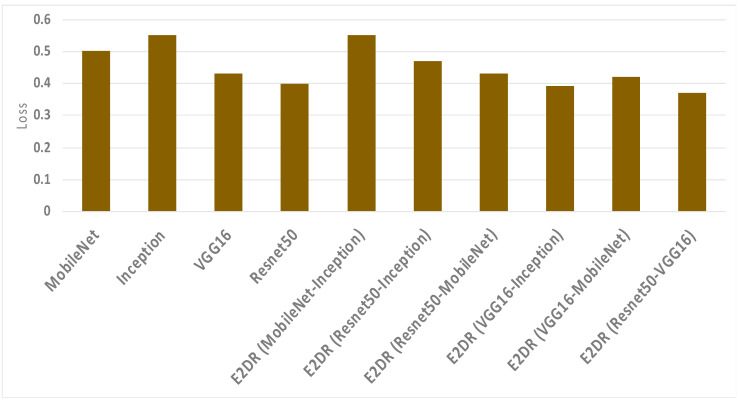
The Loss of base models and E2DR models.

**Figure 19:**
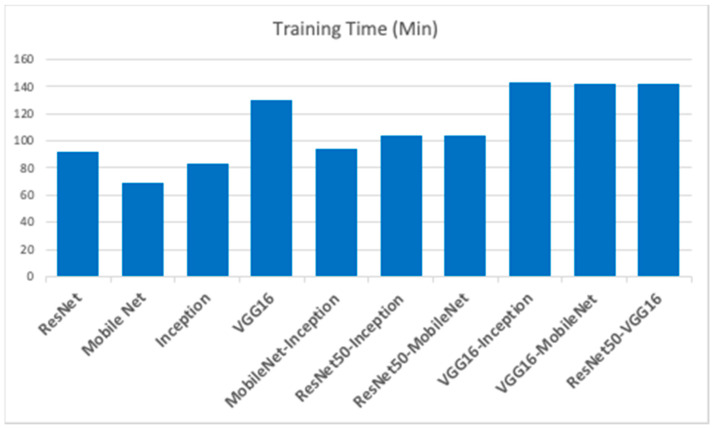
Training Time (Proposed E2DR vs. baseline deep learning models).

The results of the E2DR models showed a remarkable improvement in performance compared to the individual models. The best performing E2DR model was the stacked ensemble combination of ResNet and VGG16 with a test accuracy of 92%. The lowest-performing E2DR model with a test accuracy of 88% was the MobileNet–Inception E2DR variant, which was also expected, as the base models did not perform very well individually. The performance of each variant of the E2DR model is shown in Table 4. The improvement in accuracy was around 5–8%, which is a significant improvement considering that the base models could not exceed the late 80% in their accuracy. The fact that E2DR models reached accuracies exceeding 90% proves that the E2DR models effectively improved generalization compared to the individual base models. Other metrics, such as Precision, Recall, and F1 scores, showed similar results and improvements to accuracy, which further validates the model’s performance. The loss function used in all experiments is the Categorical Cross Entropy function, representing the confidence of predictions made by the model. The loss of the base models and the E2DR variants were in the same range with a small improvement in the E2DR models. This is because the classification confidence did not significantly improve, which means that despite making more correct classifications, which led to an increase in accuracy, the model did not have high certainty in making those predictions. Although the ensemble model in [6] achieved a higher performance with the traditional percentage-based data split, our method provides further credibility as it was tested on completely new data, which simulates real-world scenarios. This was performed by choosing subjects (Driver IDs) that are not included in the training set when constructing the validation and test sets, allowing the model to be tested on data it had not seen before. This approach is not followed in other implementations. The batch size used when recording the training duration is 32. The performance and confusion matrix of the highest performing E2DR model, which includes ResNet50 and VGG16, is presented in Figure 14. When looking at the performance report of this E2DR variant, the strongest classes from the ResNet50 model were 0 and 6, while the VGG16 performed best for classes 3 and 4. However, after analyzing the performance report of the E2DR model, our method combined the strong classes from each model in a single robust model. This is one of the most useful advantages of the E2DR model, where the model combines the skills and strong points of different models into one model, allowing the base models to complement each other in terms of performance. Similarly, Figure 15 shows the performance report and the confusion matrix of the lowest-performing E2DR variant that uses MobileNet and Inception as base models. Although it is the lowest-performing variant, it still showed a huge performance boost compared to the base models’ performance. The E2DR model effectively addressed the weak points of the Inception model (classes 0 and 7) and MobileNet model (classes 2 and 9) by boosting the classification performance for those classes in the E2DR model. Comparing the confusion matrices of the base models and the E2DR variants also improves classification performance, especially for class 8, where the confusion rate with class 7 has decreased compared to the base models. Figure 16, Figure 17 and Figure 18 visualize the performance evaluation across different metrics for the base models and the E2DR variants. The E2DR variants outperform the baseline models measured by the test Accuracy, Precision, Recall, F1 score, and Loss value, as shown in Figure 16, Figure 17 and Figure 18. As recorded in Equation (1), the computational time to fully develop the E2DR models would be the maximum training duration of the combined base models in addition to the overhead of concatenation and recommendation retrieval. The execution of the E2DR models after training can be applied in real time. The additional overhead in training the E2DR models is shown in Figure 19. It can be observed that there is an average overhead of 7% in using the E2DR models, as illustrated in Figure 19. However, due to the limited GPU computational power in the experimental hardware used as discussed in Section 5.2., we anticipate that this overhead will be significantly reduced if additional GPUs are used in the training phase. Using the baseline models, the recognition time of one image is 14.45 ms on average with 15.21 ms (on average) when using the ensemble E2DR models.

## 6. Conclusions and Future Directions

This paper examines different deep learning classification models for distracted driver classification [55,56,57,58,59] and proposes a model that improves performance and provides recommendations. We explored the performance of different models: ResNet50, VGG16, MobileNet, and Inception. All models provided viable means in detecting distracted driver actions. This paper proposes E2DR, a new model that uses stacking ensemble methods to improve accuracy, enhance generalization and reduce overfitting. Additionally, a set of recommendations are added by the model. The highest performing E2DR variant, which included the ResNet50 and VGG16 models, achieved an accuracy of 92%, while the highest performing single model was the ResNet50 with 88% accuracy. The lowest-performing E2DR model was the MobileNet–Inception variant, which achieved an accuracy of 88%, and the lowest-performing individual model was the MobileNet, with an accuracy of 82%. The accuracy difference between the highest and lowest performing models for the E2DR models and the individual models shows a significant increase in performance when using our proposed E2DR model. Other metrics were recorded and presented to evaluate the classification performance of the tested base models and E2DR variants such as Recall, Precision, and F1 score, which showed a similar increase in performance. Furthermore, the performance reports and confusion matrices showed that the E2DR models effectively addressed the weak points of the base models and boosted their classification performance. The computational complexity when developing the E2DR models from scratch is considered a limitation. Since computational speed is important in real-time applications, a light model such as MobileNet can be integrated with ResNet50 or VGG16, which in our experiment showed a significant boost in performance without adding much computational complexity.

For future work, the performance of more than two models combined in the stacking ensemble method can be examined; it was infeasible to test multiple combinations with the limited computational resources used to conduct the experiments. Furthermore, the model can be associated with the police departments to fine violators and identify drivers’ actions in case of accidents. The model can also be integrated with face recognition and alarm systems [60,61] capabilities that can allow the model to be used in a wide range of applications, such as driver authentication and theft prevention. Finally, the model can be developed and used in autonomous vehicles to detect critical conditions or situations that might endanger the driver’s health and safety, such as strokes, heart attacks, and other sicknesses. The model can recommend the vehicle to ensure the safety and health of the driver and others.

## Figures and Tables

**Table 1:** Comparative Analysis of Driver Distraction detection systems.

Paper	Model (Type)	Dataset	Validation	Pros	Cons
[7]	Deep learning Gaze estimation system	Driver Gaze in the Wild dataset	Accuracy	High performance	It can be only accurate to an extent
[8]	Deep learning Gaze estimation driver-assistant system	DR(eye)VE dataset	Ground truth	Provide suggestion	Driver gaze is subjective
[9]	Deep learning distracted driver detection	Distracted driver dataset	Accuracy, Recall, Precision, F1 score	Computationally efficient	Few epochs for training
[10]	Hierarchical, weighted random forest (WRF) model	The Keimyung University Facial Expression of Drivers (KMU-FED) and the Cohn-Kahnde datasets	Accuracy	Requires low amount of memory and computing operations	Not accurate when the face is rotated
[11]	Driver distraction detection using CNNs	State farm dataset and the AUC distracted driver dataset	Accuracy, sensitivity	Computationally efficient	Not enough validation metrics
[12]	Deep learning distracted driver detection using pose estimation	AUC Distracted Driver Dataset	Accuracy, Fl score	Pose estimation improves accuracy	Low-resolution images affected training
[13]	Driver action recognition using R-CNN	images of different driver actions	Accuracy and log loss	Effective feature representation	Small dataset
[14]	Driver Distraction recognition Using Octave-Like CNN	Lilong Distracted Driving Behavior data	Accuracy, training duration	Lightweight network	Not enough validation metrics
[15]	Temporal–Spatial Deep Learning driver distraction detection	EEG signals from 24 participants	Precision, Recall, F1 score	Unique approach	Drivers’ individual differences need to be considered
[16]	Optimized Residual Neural Network Architecture	The State Farm Distracted Driver dataset	Accuracy, training time	Enhanced model	Only detects head movement
[17]	Wearable sensing and deep learning driver distraction detection	Wearable sensing information from 20 participants	Recall, Precision, F1 score	Good potential	Small dataset
[18]	Hybrid Distraction detection model using deep learning	State farm dataset	Accuracy	Computationally expensive	Not enough validation
[19]	Triple-Wise Multi-Task Learning	AUC Distracted Driver Dataset	Accuracy, sensitivity	High detection accuracy	High computational cost
[20]	Safety-critical events prediction	Driving events from 3500 drivers	Accuracy, Recall, Precision, F1 score	Can detect potential car accidents	Hard to get enough data
[21]	CNN driver action detection system	10 drivers’ data with driving activities	Accuracy	Accurate	It does not detect the driver action
[22]	CNN driver action detection system	Distracted driver dataset	Accuracy and loss	Computationally simple	Not enough training
[23]	Distracted driver behavior detection using deep learning	Self-built dataset of drivers making phone calls and smoking	Recall, Precision, Speed	Real-time	Only trained to detect 2 driver actions
[24]	hybrid driver distraction detection model	(AUC) Distracted Driver Dataset	Accuracy and loss	High Performance	Complex
[25]	Driver Inattentiveness detection	NA	Accuracy	Comprehensive analysis of deep learning models	Not effective in detecting aggressive behavior
[26]	Deep learning manual distraction detection model	106,677 frames extracted from a video that was taken from 20 participants in a driving simulator	Accuracy, Recall, Precision, F1 score	Novel approach	Only detects manual distraction

**Table 2:** Recommendations for distracted drivers.

Class Number	Class	Recommendation
C0	Safe driving	-
C1	Texting—Right	“Please avoid texting in all cases or make a stop”
C2	Talking on the phone—Right	“Please use a hands-free device”
C3	Texting—Left	“Please avoid texting in all cases or make a stop”
C4	Talking on the phone—Left	“Please use a hands-free device”
C5	Adjusting Radio	“Please use steering control”
C6	Drinking	“Please keep your hands at the steering wheel or make a stop”
C7	Reaching Behind	“Please keep your eyes on the road make a stop”
C8	Hair and Makeup	“Please make a stop”
C9	Talking to passenger	“Please keep your eyes on the road while talking”

**Table 3:** Deep learning image classification models performance.

Model	Training Accuracy	Validation Accuracy	Test Accuracy
ResNet	0.89	0.88	0.88
VGG16	0.94	0.86	0.87
Mobile Net	0.88	0.84	0.82
Inception	0.83	0.84	0.83

**Table 4:** E2DR models performance on the test set.

E2DR Model	Accuracy	Precision	Recall	F1 Score	Loss
MobileNet–Inception	0.88	0.89	0.88	0.88	0.55
ResNet50–Inception	0.88	0.89	0.88	0.88	0.47
ResNet50–MobileNet	0.90	0.91	0.9	0.9	0.43
VGG16–Inception	0.90	0.91	0.9	0.9	0.39
VGG16–MobileNet	0.91	0.92	0.91	0.91	0.42
ResNet50–VGG16	0.92	0.92	0.92	0.92	0.37

## Data Availability

The State Farm Distracted Drivers dataset can be accessed in [47].
